# Stem cell and CRISPR/Cas9 gene editing technology in Alzheimer’s disease therapy: from basic research to clinical innovation

**DOI:** 10.3389/fgeed.2025.1612868

**Published:** 2025-08-26

**Authors:** Cong He, Baojiang Chen, Ciai Yan, Xiaoqing Zhou

**Affiliations:** ^1^ Second Clinical Medical College, Heilongjiang University of Chinese Medicine, Harbin, China; ^2^ First Clinical Medical College, Heilongjiang University of Chinese Medicine, Harbin, China; ^3^ Beijing University of Chinese Medicine Shenzhen Hospital (Longgang), Shenzhen, China

**Keywords:** Alzheimer’s disease, stem cell therapy, gene editing technology, neuroinflammation, personalized medical treatment

## Abstract

Alzheimer’s disease (AD), a progressive neurodegenerative disorder characterized by Aβ plaques, tau protein neuronal fiber tangles, and neuroinflammation, poses a significant global health problem, and current therapies focus on the symptoms rather than the cause. This paper gives a new multidimensional therapeutic form to AD treatment by exploring the integrated application of stem cell therapy and CRISPR/Cas9 gene editing technology. The study comprehensively dissected the roles of neural stem cells (NSCs), induced pluripotent stem cells (iPSCs) and mesenchymal stem cells (MSCs) in neural replacement, neuroinflammation modulation and neuroplasticity enhancement, and also explored the application of CRISPR/Cas9 in modifying the pathogenic variants of AD-related genes (APP, PSEN1 and PSEN2). The key findings suggest that gene-edited iPSCs can reduce abnormal Aβ and tau protein accumulation in AD models, improve cognitive function, and provide a platform for disease modeling and drug screening. Stem cell transplantation promotes neurogenesis and synaptic plasticity by secreting neurotrophic factors to improve the brain microenvironment. Despite the challenges of off-target effects, immune rejection, and long-term safety, the synergistic application of these two technologies offers a breakthrough solution for AD treatment. This paper highlights the translational potential of combining stem cells with gene editing technology, which is expected to drive clinical applications in the next 5–10 years. The integration of these advanced technologies not only addresses the limitations of current AD treatments, but also paves the way for a personalized medical approach that is expected to revolutionize the AD treatment landscape and bring new hope to patients worldwide.

## 1 Introduction

Alzheimer’s disease (AD) is a progressive neurodegenerative disease, and the epidemiological trend of AD shows a continuous increase in prevalence and incidence in the context of global population aging. According to predictions the number of AD patients worldwide will reach about 150 million by 2050 (Arayici and Kose, 2025; Beamon et al., 2025). The roots of the disease are more complex on a pathologic level, with the deposition of Aβ plaques being one of the important pathologic features; Aβ is produced by the cleavage of amyloid precursor proteins by β- and γ-secretase enzymes. Aβ42 has strong aggregation properties, and when it accumulates in excess, neurotoxicity occurs, with abnormal phosphorylation of tau proteins leading to microtubule depolymerization, and coexistence of neuronal dysfunction and death in neurogenic fibril tangles (Bermudez et al., 2025). Neuroinflammation is also important in the pathogenesis of AD, as microglia and astrocytes are activated to release a number of proinflammatory factors and chemokines, which can exacerbate neuronal damage (Chasse et al., 2025). Genetic factors also play an important role in the pathogenesis of AD. Mutations in the APP, PSEN1, and PSEN2 genes trigger Familial Alzheimer’s Disease (FAD), also known as early-onset AD, which accounts for about 5%–10% of all AD patients. Late-onset AD, on the other hand, is caused by many genetic and environmental risk factors, in addition to the APOEε4 allele, there are other genetic risk factors, TREM2, CD33, ABCA7, and others (Vance et al., 2024; Valdes et al., 2025). These risk factors are mainly expressed in microglia, which also play an important role in neuroinflammation. When microglia are activated, they produce more pro-inflammatory factors, which further exacerbate neuronal damage, and thus microglia are a major target for research on the pathogenesis of AD.

There are now pharmacologic and non-pharmacologic treatments for AD, both of which have limitations. Medications, donepezil, and carboplatin are cholinesterase inhibitors that can help patients improve their cognitive abilities, but only reduce the disease, not stop it from progressing (Tang et al., 2025; Tripathi et al., 2025). Memantine is an NMDA receptor antagonist that regulates neurotransmitter homeostasis, again without altering the disease process. Immunotherapy against Aβ and tau proteins has improved in recent years, as is the case with aducumab, but this therapy remains controversial for clinical use and its efficacy and safety will have to be reconfirmed (Yu et al., 2025). In terms of non-pharmacological interventions, cognitive training and exercise therapy can improve cognitive function and quality of life to some extent, but the effect is not obvious. Because current treatment modalities are difficult to fundamentally reverse the course of AD and most of them are only symptomatic and do not completely cure the disease, we need to find more effective treatments with a view to obtaining a better prognosis and a better quality of life for the patients (Choi et al., 2025; Xing et al., 2025).

In recent years, the rapid development of stem cell technology and gene editing technology has brought new treatment possibilities for many diseases, including AD. Human induced Pluripotent Stem Cells (hiPSCs) are highly valued for their potential to mimic AD pathology and advance drug discovery by reprogramming somatic cells from AD patients into hiPSCs, which are then grown into neurons and glial cells. Researchers have constructed *in vitro* models that can recapitulate core pathological features of AD, such as amyloid plaques and neuroprogenitor fibril tangles, and these models, created by relying on hiPSCs, have given new perspectives on probing the molecular bases of AD pathogenesis and have been a powerful force in the search for potential therapeutic targets (Mahairaki et al., 2014; Mohamet et al., 2014; Young and Goldstein, 2023). Moreover, platforms relying on hiPSCs can perform high-throughput screening of drug libraries to find compounds that can cut Aβ formation or induce Aβ clearance, which can accelerate the emergence of new drugs. Related studies have shown that screens relying on hiPSCs have identified a number of small molecules that can modulate β- and γ-secretase activity and thereby cut down on Aβ formation (Bahnassawy et al., 2024; Gallo et al., 2024). These developments show the great importance of hiPSCs technology in deepening our understanding of AD pathogenesis and creating new therapies.

## 2 Application of stem cell therapy in AD

### 2.1 Types of stem cells and their roles in AD

There are many therapeutic potentials of Neural Stem Cells (NSCs) in the treatment of AD, with the main advantage being the ability of neural replacement and repair. NSCs of embryonic or adult origin can differentiate into neurons, astrocytes, and oligodendrocytes, which fill in the neuronal cells lost during AD pathology, and secrete brain-derived neurotrophic factor (BDNF) and glial cell-derived neurotrophic factor (GDNF), which improves synaptic plasticity, supports the survival of pre-existing neurons, and maintains function (Pecoraro et al., 2025; Wu H. et al., 2025). In recent years, studies have shown that the gene editing ability of NSCs provides an important platform for the study of AD pathomechanisms, and potential therapeutic targets can be screened by editing AD risk genes, such as APP and PSEN1, and mimicking the process of β-amyloid deposition and abnormal phosphorylation of tau proteins in mouse and human cell models (Yeapuri et al., 2025). However, the clinical translation of NSCs runs into two bottlenecks: a survival rate of less than 30% after transplantation because the ischemic and hypoxic microenvironment triggers apoptosis, and the risk of immune rejection, with the implementation of prolonged immunosuppression (Gowrishankar et al., 2024; Wang et al., 2024). Recent studies have shown that pre-differentiation of NSCs into neuronal precursor cells or incorporation of anti-apoptotic factors can improve survival, but long-term efficacy has yet to be demonstrated (Tang et al., 2024).

Induced Pluripotent Stem Cells (iPSCs) are obtained after reprogramming of somatic cells, and they have a variety of differentiation possibilities to turn into some cell types related to AD therapy, such as neurons, neural progenitors, and microglia (Liu et al., 2025). Its core value is reflected in disease modeling and personalized treatment. reprogramming fibroblasts from AD patients into iPSCs, which then differentiate into neurons, will be able to recapitulate typical pathological phenomena such as Aβ plaque formation, tau tangles, and so on, *in vitro*, and give a precise model for drug screening (Schulz, 2021; Yefroyev and Jin, 2022). The SALL4 single-factor reprogramming technology developed by the Guangzhou Health Research Institute in 2024 reprogrammed mouse fibroblasts into iPSCs, and these iPSCs were karyotypically normal, and this technology also markedly improved the induction efficiency of iPSCs, which demonstrates the important role of SALL4 in the reprogramming process (Xiao et al., 2024). And another study on bionic scaffolds shows the breakthrough of this technology by Yongxiang Jiang’s team, which demonstrated that the bionic scaffold technology can directionally induce iPSCs to differentiate into pigment-free ciliary epithelial-like cells, repair lens suspensory ligament fibers and improve their mechanical properties in an EL rabbit model (Chen et al., 2025). However, iPSCs-derived neurons still have some differences in the reproducibility of AD pathology, with approximately 20% of clones showing tau hyperphosphorylation, which needs to be improved with the help of epigenetic modulation (Ochalek et al., 2017).

Mesenchymal Stem Cells (MSCs) belong to a group of pluripotent stromal cells that can be isolated from different tissues, like bone marrow, adipose tissue, and umbilical cord blood, and these cells are able to differentiate into cells of the mesodermal lineage, that is, into osteoblasts, chondrocytes, and adipocytes, although they can also give rise to neuronal cells in some cases (George et al., 2019). Because of its strong immunomodulatory ability to secrete neurotrophic factors and move toward the site of brain injury, MSCs have particular advantages in the treatment of AD (Hernandez and Garcia, 2021). Its main mechanisms are the inhibition of microglia overactivation through the IDO and PGE2 pathways, induction of an M2-type anti-inflammatory phenotype, and secretion of exosomes carrying factors such as miR-124 to promote endogenous neural stem cell proliferation (Zheng et al., 2015; Han et al., 2022). Relevant studies have shown that the APOs@BP nanosystems developed by Zhou’s team are able to achieve highly efficient gene transfection in serum-containing environments, with a transfection efficiency as high as about 73.9%, far exceeding that of other high molecular weight cationic transfection agents. By loading miR-124 and all-trans retinoic acid derivative (atRAN) into MSCs, the potential of neural differentiation was activated to direct neurogenesis, thereby improving cognitive function in AD model mice (Jin et al., 2025). A phase I/II clinical trial (NCT04388982) showed that after 12 weeks of intranasal delivery of gene-edited MSCs exosomes in patients with mild-to-moderate AD, the AD Assessment Scale-cognitive component (ADAS-cog) scores in the medium-dose group decreased by 2.33 points from baseline, the Montreal Cognitive Assessment scores improved by 2.38 points from baseline, and the ADAS-cog scores at 36 weeks decreased by 2.33 points from baseline by 3.98 points, suggesting improved cognitive function. Meanwhile, the hippocampal atrophy in patients in the medium-dose group was relatively small, but did not reach statistical significance. There were no adverse events during the entire treatment period and during the follow-up period, indicating that intranasal delivery of human adipose mesenchymal stem cell-derived exosomes is safe and well tolerated in the treatment of AD (Xie et al., 2023). Compared with NSCs and iPSCs, MSCs have obvious advantages. MSCs can be easily obtained from adult tissues such as fat and bone marrow, avoiding ethical problems (Gopalarethinam et al., 2023). The immunogenicity of MSCs is low, allogeneic transplantation does not need matching, and in most clinical studies, the safety of MSCs transplantation is equivalent to that of placebo (Berglund et al., 2017). In addition, MSCs have the characteristics of self-renewal, multi-directional differentiation, immunomodulation and anti-inflammation, and they also play a role in the field of regenerative medicine, and their tumorigenicity is low, which improves the clinical safety (Thate et al., 2021). On the basis of optimizing the delivery system of CRISPR-Cas9 RNP, Han et al. efficiently knocked out the β2 microglobulin (B2M) gene of human umbilical cord mesenchymal stem cells (UC-MSCs) (editing efficiency >85%). The MHC I molecules on the surface of B2M−/− MSCs were almost undetectable, which significantly prolonged their survival time when co-cultured with CD8 T cells, and inhibited the proliferation of CD8 T cells to less than 35% of the control group. After IFN-γ pretreatment, the immunoregulatory factors IDO-1 and PGE2 secreted by B2M−/− MSCs increased significantly, further enhancing the immunosuppressive activity. These results indicate that the immune escape and immunomodulatory function of MSCs can be improved by RNP-mediated B2M knockout, which provides a new strategy for its application in allogeneic cell therapy (Han et al., 2024). In Parkinson’s disease (PD) model, Lee et al. edited umbilical cord blood mesenchymal stem cells by CRISPR/Cas9 technology to make them secrete soluble RAGE (sRAGE), and then transplanted these cells into the striatum of rotenone-induced PD model mice. It was found that these cells could reduce the death of nerve cells by inhibiting AGE-albumin, and the motor ability of mice was also improved. The secretory characteristics of sRAGE secreted UCB-MSC also make it have certain advantages in playing a therapeutic role, which provides a new idea for the treatment of neurodegenerative diseases (Lee et al., 2019). MSCs edited by CRISPR also performed well in cardiovascular disease research. Studies have shown that editing the TLR4 gene of human bone marrow mesenchymal stem cells makes them lose the ability of inflammatory response, and injecting the edited cells into the myocardial infarction area of mice with myocardial infarction. After 4 weeks, the survival rate of mice is improved, the left ventricular remodeling and cardiac function are improved, and the edited cells that survive in the infarcted area form myocardial islands, which reduces the inflammatory response and fibrosis. This example not only shows the function enhancement effect of MSCs under CRISPR editing, but also verifies the clinical safety characteristics of its low inflammatory reaction (Schary et al., 2023). In the treatment of spinal cord injury (SCI), various stem cell types show different therapeutic potentials. Umbilical cord mesenchymal stem cells can reduce neuronal apoptosis, inhibit glial scar formation, improve motor function, and show good therapeutic effects in animal experiments. MSCs derived from fat also improve the functional recovery after SCI by secreting neurotrophic factors and promoting nerve regeneration. These studies show that the therapeutic effect of stem cells can be further improved through gene modification or combined therapy (Huang L. et al., 2021). Future research can also try combined therapy, such as combining exosomes derived from MSCs with anti-Aβ antibodies, which can achieve the purpose of removing pathological proteins, and at the same time protect and repair brain structures by using the neuroprotective and regenerative effects of MSC-exosomes. With the continuous exploration of the therapeutic potential of MSC-exosomes and the advancement of clinical trials, MSC-exosomes is expected to become the first approved regenerative medicine therapy for AD, thus realizing a new therapeutic paradigm from “fighting pathology” to “ecological restoration” (Chen et al., 2021) (Table 1).

**TABLE 1:** The potential, challenges, and future research directions of different stem cell types in AD treatment. It summarizes the core value and mechanisms of action, bottlenecks and challenges, and future research directions for NSCs, iPSCs, and MSCs in AD treatment.

Stem cell type	Core value and mechanism of action	Bottlenecks and challenges	Future research directions
NSCs (Wang et al., 2024)	Neural replacement and repair, genetic editing aids pathogenesis research	Low survival rate, immune rejection	Pre-differentiation or using anti-apoptotic factors to improve survival rate
iPSCs (Schulz, 2021)	Disease modeling and personalized treatment, reprogramming technology enhances induction efficiency	Derivative neuron pathological feature reproducibility differences	Epigenetic regulation optimizes pathological feature reproducibility
MSCs (Chen et al., 2021)	Immune regulation, secretionof neurotrophic factors, exosomes promote neural repair	Some clinical efficacy not statistically significant	Combined therapy strategies, advancing clinical trials

### 2.2 Mechanisms of stem cell action

NSCs can secrete neurotrophic factors such as BDNF to promote neuronal survival and improve neuroplasticity. In the APP/PS1 transgenic mouse model, transplantation of primary NSCs, whether from embryonic or adult neural tissue sources, was effective in increasing BDNF levels in the hippocampal region, promoting neurogenesis, and improving cognitive function (Li et al., 2016; Chen et al., 2017). The molecular mechanism is that BDNF binds to TrkB receptors on the surface of neurons and upregulates the expression of the postsynaptic dense protein PSD-95 via the PI3K-AKT pathway on the one hand; on the other hand, it promotes the proliferation of neural progenitor cells via the MAPK pathway (Yoshii and Constantine-Paton, 2014; Numakawa et al., 2018). It has been shown that human neural stem cell transplantation in the APP/PS1 mouse model increases synaptic density and improves neurometabolic activity to enhance cognitive function, which may be related to the secretion of neurotrophic factors by the transplanted cells and the promotion of synaptic growth and neuroplasticity (Li et al., 2016). The mechanism of action of NSCs also includes anti-inflammatory effects, i.e., inhibition of microglia overactivation through secretion of anti-inflammatory factors, which reduces the release of pro-inflammatory cytokines and attenuates neuroinflammation (Zhou et al., 2022). It also promotes synaptic remodeling, as evidenced by the upregulation of synaptophysin and the expression of the postsynaptic density protein PSD-95, which promotes an increase in synaptic density and optimizes the connectivity of neural networks (Xiong et al., 2018).

IPSCs have multidirectional differentiation potential and can differentiate into neurons in a targeted manner to replace damaged cells, which is a unique advantage in constructing disease models. The iPSC-derived neurons obtained with reprogrammed AD patient fibroblasts can mimic the pathological phenomena of AD, like Aβ deposition and tau protein phosphorylation, thus giving a platform to probe the disease mechanism and screen drugs (Sharma et al., 2020). The potential for personalized therapy encompasses flexibility and controllability, with the gene editing technology CRISPR/Cas9 to accurately correct disease-causing mutations, enhance cellular stability, and strengthen immunocompatibility (Shtrichman et al., 2013). Autologous iPSC-derived cells do not produce immune rejection, but there is a risk of genetic mutations associated with the reprogramming process, including point mutations and chromosomal abnormalities (Howden et al., 2018). At this stage, the clinical translation of iPSCs still suffers from technical obstacles such as genetic instability and cellular heterogeneity. The transient deletion of the G1/S cell cycle checkpoint during reprogramming induces genetic mutations, some of which overlap with cancer-related signaling pathways, and the epigenetic differences of different iPSC clones affect the differentiation efficiency and therapeutic consistency (Yoshihara et al., 2017).

**FIGURE 1:**
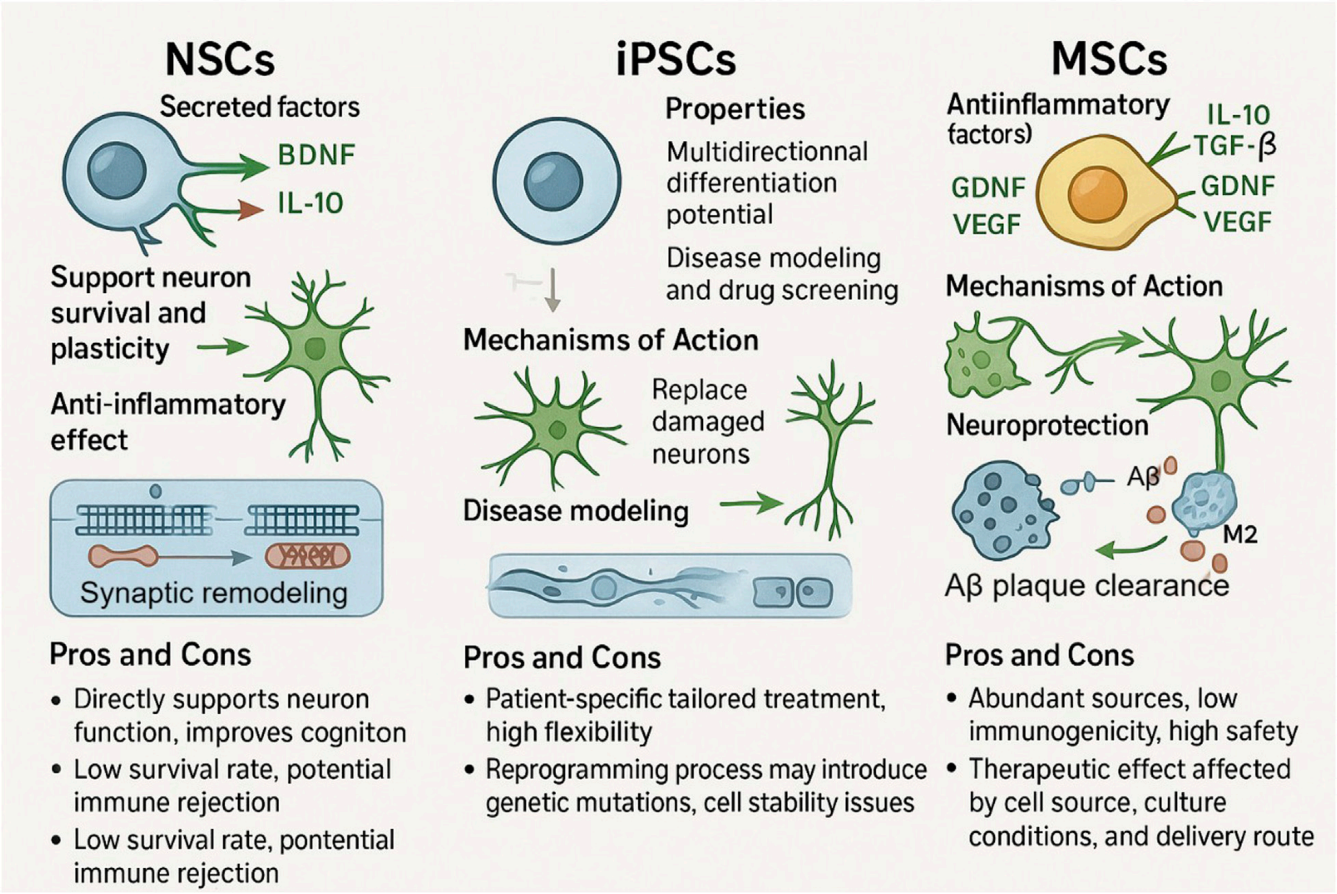
Functions, mechanisms, and pros and cons of NSCs, iPSCs, and MSCs. NSCs can maintain neuronal survival and enhance plasticity by secreting neurotrophic factors (BDNF, IL - 10), in addition to anti-inflammatory capabilities, although it has a relatively low survival rate and the potential for immune rejection. iPSCs are capable of differentiating in many directions and show some potential for constructing disease models and performing drug screening, yet sudden genetic mutations may occur during reprogramming and cell stability is difficult to ensure. MSCs rely on the secretion of anti-inflammatory factors such as TGF-β and IL-10 and the removal of Aβ plaques for neuroprotection. MSCs are a rich source of cells with low immunogenicity, but their therapeutic efficacy is limited by the source of the cells, the culture conditions, and the route of delivery.

MSCs mainly function through two mechanisms, paracrine and immunomodulation, and their main mechanism for the treatment of AD is anti-inflammatory and immunomodulation, which can secrete anti-inflammatory factors such as IL-10 and TG-β, inhibit microglia polarization to pro-inflammatory M1 type, promote microglia polarization to anti-inflammatory M2 type, and enhance microglia phagocytosis and clearance of Aβ plaque, and they can also secrete GDNF, VEGF and other factors that promote neuronal survival, angiogenesis, and synaptic remodeling (van Buul et al., 2012; Li et al., 2020; Yang et al., 2020). The bone marrow MSCs exosome miR-146a regulates the inflammatory response in diabetic retinopathy by mediating the TLR4/MyD88/NF-κB pathway and decreasing the levels of inflammatory factors such as TNF-α (Gu et al., 2022). In phase I clinical trials, intracerebroventricular injection of human umbilical cord-derived MSCs has been shown to be safe and well tolerated in patients with mild-to-moderate AD, attenuating cerebral atrophy and improving patients’ cognitive profiles (Kim et al., 2021). However, MSCs themselves are characterized by low immunogenicity, and their efficacy is still subject to numerous factors such as route of administration, dose optimization, culture conditions, and so on (Chang et al., 2013; Lublin et al., 2014; Chen et al., 2023; Shan et al., 2024). Intravenous infusion is relatively simple to perform, although cells are easily retained in the lungs (Shan et al., 2024); while intracerebral or intrathecal injections, although they can elevate the local concentration of cells, should be considered for their invasive risks (Mesa Bedoya et al., 2024) (Figure 1).

### 2.3 Clinical and experimental research progress

In stem cell therapy research for AD, clinical and experimental studies are developing rapidly and with great potential. It has been shown that in the APP/PS1 mouse model, transplantation of NSCs resulted in a dramatic increase in BDNF levels, a promotion of hippocampal nerve growth, and an improvement in cognitive function (Li et al., 2016; Zheng et al., 2017). MSCs reduce neuroinflammation by secreting anti-inflammatory factors and promote neuronal survival and angiogenesis by secreting neurotrophic factors in a paracrine manner. After transplantation of MSCs in the lateral ventricles of 3xTg-AD model mice, working memory remained unchanged, Aβ levels decreased, and neural neogenesis increased (Karvelas et al., 2022). It has been shown that MSCs secrete anti-inflammatory microRNAs (miRNAs), activate microglia, and reduce oxidative stress, and these mechanisms have been associated with improved cognitive function in AD mice. MiR-146a secreted by MSCs inhibits neuroinflammation, and miR-124 contributes to neuronal maturation and functional recovery (Nakano et al., 2020; Chen et al., 2021). Stem cell therapies have shown positive results in clinical trials, with Longeveron’s Laromestrocel demonstrating a favorable safety profile and preliminary efficacy in a Phase 2a clinical trial in patients with mild AD, which improved cognitive functioning and life-scenario abilities when used for atrophy of the whole brain or part of the brain region (Rash et al., 2025). Other clinical trials have also shown that stem cell therapies are both safe and effective, and that genetically corrected iPSCs as well as mouse models are uniquely suited for evaluating and managing AD pathology when compared to traditional pharmacologic interventions such as aducumab. Aducarmab, a monoclonal antibody specifically targeting Aβ plaques, has shown more controversial results in clinical trials, and while it was able to reduce Aβ aggregation, the effects on synaptic function and cognitive improvement were nevertheless mild and inconsistent (Haddad et al., 2022). In contrast, iPSCs obtained from AD patients and genetically edited to correct pathogenic variants in genes such as APP or PSEN1 reduced Aβ production and improved synaptic density and function after transplantation into AD mouse models. It was demonstrated that editing iPSCs to transform the APOE4 allele to APOE3 resulted in reduced tau protein phosphorylation and Aβ secretion as well as improved synaptic plasticity and cognitive function (Najm et al., 2020). Similarly, CRISPR/Cas9 correction of the APP mutation resulted in reduced Aβ deposition and improved synaptic connectivity, which was superior to what is usually observed with aducumab-like therapies in terms of overall pathologic reversal (Yin et al., 2014).

### 2.4 Safety and ethical considerations

Although stem cell therapy has great potential for the treatment of AD, there are many difficult aspects of stem cell therapy; embryonic stem cells are involved in the generation of life and ethical dilemmas, there are fewer sources of embryonic stem cells, and there is a lower rate of survival when transplanted into someone else’s organism (de Peppo and Marolt, 2012). Although iPSCs avoids the problem of immune incompatibility, the technique is less efficient and carries a risk of tumorigenicity (Karami et al., 2022). Immune rejection may also occur after stem cell transplantation, which may have an impact on the survival and function of the transplanted stem cells, and the long-term effects of stem cells cannot be confirmed and need to be examined over a longer period of time with follow-up. Stem cell therapy has been used in the treatment of AD, and some studies have shown that MSCs have shown potential to reduce brain atrophy in clinical trials, and neural stem cell transplants have shown some efficacy in animal models, but more research is needed to achieve widespread use, including technological improvements, safety evaluations, and ethical considerations (Hoveizi et al., 2018; Rash et al., 2025).

## 3 Application of gene editing technology in AD

### 3.1 Integration of CRISPR/Cas9 technology in AD research

The principle of CRISPR/Cas9 gene editing technology originates from the bacterial coping mechanism against viral infection. When a virus invades a bacterium, a fragment of viral DNA is incorporated into the bacterium’s CRISPR sequence, forming a guide RNA (gRNA), which combines with the Cas9 nuclease to cut and localize the viral DNA (Gebre et al., 2018). In scientific research, researchers have used this principle to artificially synthesize gRNAs that are specific to the location of a target gene, and then direct the Cas9 nuclease to cut it, and the cell then relies on its own DNA remediation mechanism to process the cuts, resulting in precise modifications such as knockouts, insertions, or substitutions (Bannikov and Lavrov, 2017; El-Mounadi et al., 2020). In AD research, many CRISPR/Cas9 systems have been tested in mouse models and iPSCs, and there are newer variants of the traditional CRISPR/Cas9 system, like base editing and Prime editing included, such as in mouse models, where CRISPR/Cas9 has been used to accurately knock-in and knock-out AD-related genes (e.g., APP and PSEN1), which results in a model that more closely resembles the human AD pathological condition. Researchers have created mouse models with mutations in specific AD-related genes (e.g., APP and PSEN1) that exhibit the key pathologies of AD, amyloid plaques and neuroprogenitor fibrillary tangles, which endow a critical means of delving deeper into the mechanisms of disease onset and diagnostic approaches (Yeapuri et al., 2025). CRISPR/Cas9 in iPSCs has been used to correct disease-causing mutations, as exemplified by turning the APOE4 allele into APOE3, which has been shown to cut down on AD-related phenotypes, tau protein phosphorylation and Aβ secretion included (Schmid et al., 2020). Base editing techniques have been employed to add protective mutations without causing double-strand breaks, by adding the A673T mutation to the APP gene, which reduces the chances of off-targeting and thus improves safety (Guyon et al., 2021). CRISPR/Cas9 technology has shown clear potential for creating AD mouse models that can highly mimic human AD pathology, and has been used to precisely control pathological processes such as Aβ formation and tau protein phosphorylation, shedding light on potential therapeutic approaches. Recent studies have shown that cutting down Aβ production or enhancing Aβ clearance by gene editing can significantly improve cognitive function and cut down neuroinflammation in AD models, and also normalize tau protein phosphorylation levels, which in turn improves cognitive deficits and attenuates other AD-associated pathological alterations (Chacko et al., 2023; Tripathi et al., 2024). The clinical application of CRISPR/Cas9 technology in AD research faces a number of serious challenges, one of which is the off-target effect. GRNAs may be partially complementary to non-targeted DNA sequences, which may induce the Cas9 nuclease to perform cleavage operations at unintended sites, a scenario that may lead to potential mutations with unpredictable biological consequences and an increased risk of tumorigenesis (De Plano et al., 2022). In addition, the immune response cannot be ignored, as foreign Cas9 proteins and gRNAs may stimulate the immune system, which may interfere with the editing process and cause adverse effects, and the special physiological barriers of the CNS and the complexity of the cell types make it very difficult to successfully deliver the CRISPR/Cas9 system to patients with AD (Hanafy et al., 2020). Delivery of CRISPR/Cas9 components, which is a current problem, is being attempted with many different methods of delivery, one of which is adeno-associated viruses (AAVs), which are capable of efficiently delivering neurons and glial cells, and which also do not integrate into the genome of the host cell, thereby reducing off-target effects (Haggerty et al., 2020; Challis et al., 2022). The other is lipid nanoparticles, which can be used as a non-viral alternative to deliver CRISPR components; they can protect CRISPR from degradation and also help cross the blood-brain barrier; nanoparticles, too, can encapsulate CRISPR/Cas9 components against degradation and allow CRISPR/Cas9 components to cross more smoothly through the multiple membranes into neurons (Wang et al., 2025; Wu F. et al., 2025). Also, it is possible to use a cell-penetrating peptide to guide CRISPR/Cas9 into the cell, and it's kind of hard to edit *in vivo* for non-dividing cells like neurons, which have low metabolic activity and no DNA repair mechanisms. But base editing and Prime editing technologies may have potential, and these new gene editing methods allow precise single-base changes to be made without causing double-strand breaks, which could reduce the risk of unintended mutations and make *in vivo* applications a bit safer (Ramakrishna et al., 2014; Mesaki et al., 2023).

### 3.2 Advantages and limitations of gene editing

CRISPR/Cas9 gene editing technology has obvious advantages in the treatment of AD. Its high specificity and efficiency make it capable of precisely editing AD-related genes and correcting the genetic errors that cause AD at the source. GRNAs are complementarily paired with the target DNA sequences, and the Cas9 nuclease, led by it, can accurately locate and cut off specific genes, and thus effectively editing those genes related to AD (Kuruvilla et al., 2018; He et al., 2025). Compared with traditional gene therapy, CRISPR/Cas9 technology has a unique advantage in creating AD models. According to studies, CRISPR/Cas9 technology has been used to successfully create mouse models with mutations in specific AD-related genes, which can effectively mimic the pathology of human AD, giving important tools to study the pathogenesis of AD as well as therapeutic approaches (D'Agostino and D'Aniello, 2017; Garcia-Agudo et al., 2024). However, CRISPR/Cas9 technology has shortcomings when it comes to AD therapy in that off-target phenomena have not been resolved, and unintended editing of non-target genes may cause unpredictable biological effects. Singh et al are working on the development of more sensitive off-target predictors and detectors, and are also utilizing molecular engineering to improve the specificity of the CRISPR editing tool (Singh et al., 2015; Kamli and Khan, 2025). Gene delivery systems also present many challenges, and delivering the CRISPR/Cas9 system efficiently and safely to target cells in the central nervous system is one of the major challenges in reaching clinical applications. Researchers are currently exploring a variety of delivery methods, such as viral vectors, non-viral vectors, etc., with the goal of efficiently delivering the CRISPR/Cas9 system. Ethical barriers also constrain the widespread use of CRISPR/Cas9 technology in the treatment of AD, and the ethical controversies arising from germ cell editing in particular need to be explored in depth, and relevant regulations need to be developed in parallel with the development of the technology (Yang et al., 2022; Ling et al., 2025).

### 3.3 Clinical translation prospects of gene editing technology

Gene editing technology has shown great potential for clinical translation in AD, and early clinical applications of the CRISPR/Cas9 system have brought a ray of hope for treating early AD or for preventive genetic intervention. Studies have shown that with the help of CRISPR technology, by excising the C-terminal fragment of the APP protein, APP can be prevented from being cleaved by β-secretase, which in turn reduces the production of Aβ, and also enhances the level of sAPPα (Sun et al., 2019). In mice, this editing strategy not only cut down on amyloid accumulation and associated neuroinflammatory markers, but also markedly increased the level of sAPPα, which coincides with the beneficial results generated by the APOE4-Christchurch gene mutation mimicry study, and also exhibits the possibility of serving as a potential intervention for APOE4-related AD (Sun et al., 2019).The development of gene editing technology in AD treatment depends on breakthroughs in many aspects. From the technical aspect, the CRISPR/Cas9 system should be improved to cut down the off-target phenomenon, and the improvement of editing efficiency and safety performance has become the key to improvement, and the development of a more efficient gene delivery system is also very important, especially the carrier that can effectively penetrate the blood-brain barrier, which is the key to reach the clinical application of gene editing technology in AD. The development of more efficient gene delivery systems is also very important, especially vectors that can effectively penetrate the blood-brain barrier, which is a necessary condition for the clinical application of gene editing technology in AD (Cheng et al., 2021). Moreover, an ethical framework cannot be missing, and as the technology develops and the discussion and standardization of gene editing becomes feasible, the convergence of gene editing and stem cell technology may give a comprehensive and effective approach to AD treatment, with gene editing to correct disease-causing mutated genes in the stem cells, and then implanting the stem cells into the patient, which will probably turn out to be a key step towards the future of AD treatment (Poon et al., 2017; Bhushan et al., 2024).

## 4 Future of combined stem cell and gene editing technologies in AD therapy

### 4.1 Technical background and theoretical basis

Considerable progress has also been made in utilizing CRISPR/Cas9 technology to generate stem cells for AD-related gene repair. Researchers have found that CRISPR/Cas9 has a very high accuracy rate in correcting disease-causing genes, and it has been found that converting APOE4 to APOE3 in iPSCs carrying the APOE4 allele greatly reduces the AD-related properties of the cells (Liu et al., 2024). In experiments in which the APOE4 gene was edited with CRISPR/Cas9 and converted to APOE3 in iPSCs from patients with sporadic AD, tau protein phosphorylation and ERK1/2 phosphorylation were weaker in edited neurons compared with unedited APOE4 neurons, and edited neurons showed isoform-dependent phosphorylated tau protein release decreased (Lin et al., 2018; Khan et al., 2025). In the study, after changing the APOE4 allele to APOE3/3 genotype in iPSCs from two AD patients with CRISPR/Cas9, the edited neurons did not show significant differences in Aβ42 secretion levels compared with unedited APOE3 neurons, and these data suggest that the combination of CRISPR/Cas9 with stem cells has great potential for the diagnosis and treatment of AD (Lin et al., 2018) (Table 2).

**TABLE 2:** A summary of CRISPR/Cas9 approaches that have successfully reduced Aβ/tau pathology and improved cognition in AD models.

Gene	Mutation	CRISPR editing approach	Model system	Delivery system	Outcome on Aβ/Tau	Cognitive function improvement
APP	A673T	Base editing	iPSCs	Lipid nanoparticles	Reduced Aβ	Yes
PSEN1	A79V	CRISPR/Cas9 correction	iPSCs	AAV	Reduced Aβ	Yes
PSEN2	N141I	CRISPR/Cas9 correction	iPSCs	Electroporation	Reduced Aβ	Yes
TREM2	R47H	CRISPR/Cas9 correction	iPSC-derived microglia	Lentivirus	-	Improved microglial function potentially affecting cognition
CD33	-	CRISPR/Cas9 modulation	iPSCs	AAV	-	Reduced neuroinflammation, likely beneficial for cognition

### 4.2 Successful cases and challenges

**FIGURE 2:**
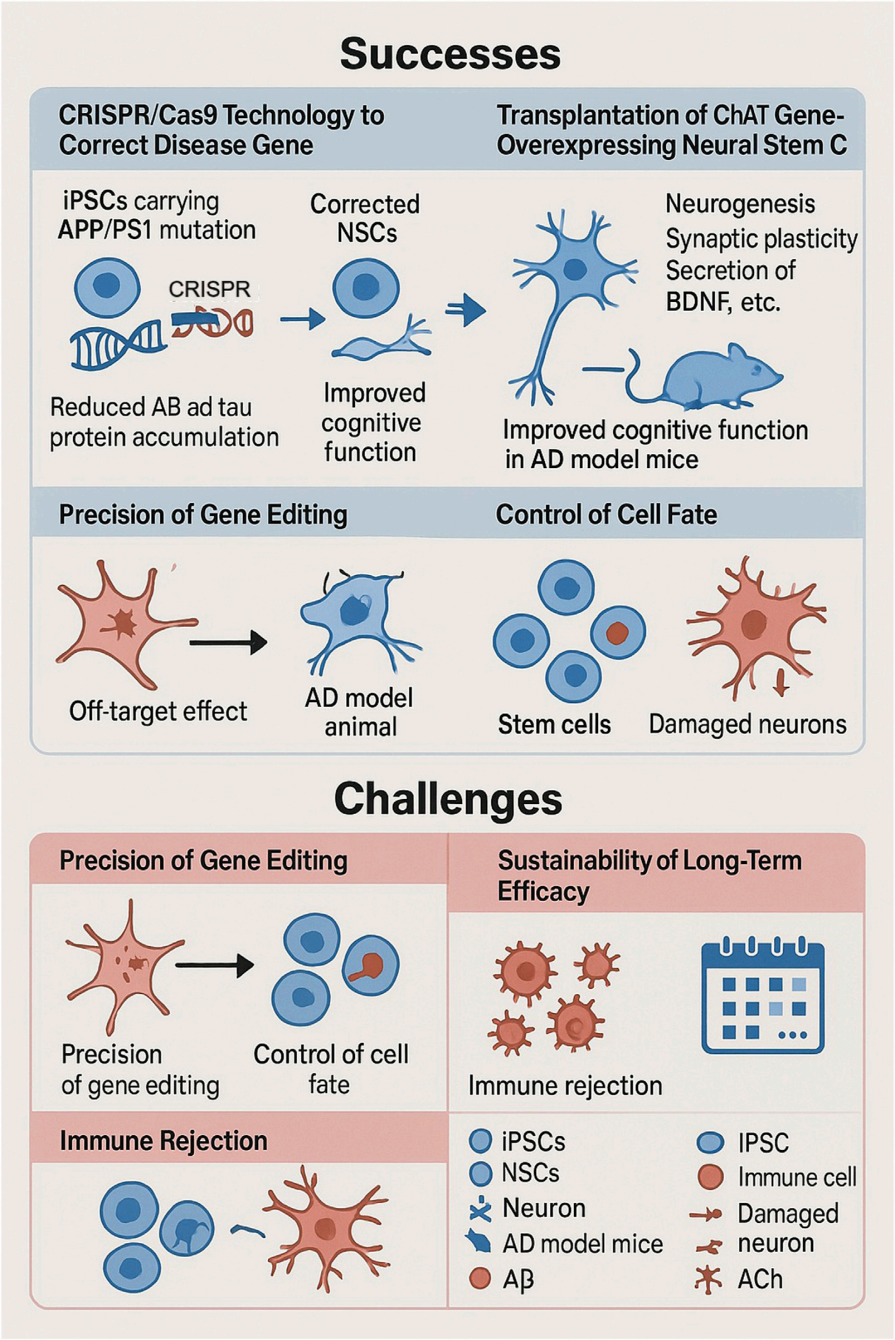
Successes and challenges of stem cell and gene editing technologies in the treatment of neurodegenerative diseases. Successes include the use of CRISPR/Cas9 to correct disease genes in iPSCs carrying APP/PS1 mutations, to reduce the accumulation of Aβ and tau proteins, and the transplantation of neural stem cells overexpressing ChAT to promote neurogenesis and improve cognitive function in AD model mice. Challenges include ensuring the precision of gene editing to avoid off-target effects, controlling cell fate to replace damaged neurons, and addressing long-term efficacy and immune rejection.

The combination of stem cell technology and gene editing technology has yielded some results in AD therapy, but many problems remain.CRISPR/Cas9 technology repaired the pathogenic mutant gene in APP/PS1 mutant iPSCs, and the abnormal accumulation of Aβ and tau protein was significantly reduced when they were re-differentiated into neural cells, which improved cognitive function in AD model mice. Neural stem cells transplanted into AD model animals promote neurogenesis, enhance synaptic plasticity and secrete neurotrophic factors such as BDNF, improving the brain microenvironment (Pourhadi et al., 2024). In another study, human neural stem cells overexpressing the ChAT gene were transplanted into APPswe/PS1dE9 mice and restored cognitive function by synthesizing acetylcholine, clearing Aβ, and neuroregenerative effects (Park et al., 2020). These success stories are a good example of the potential and advantages of combining stem cell and gene editing technologies in the treatment of AD, although there are still some challenges and the accuracy of gene editing needs to be improved.CRISPR/Cas9 technology, although highly capable of editing, is still off-target, which may result in unintended mutations in other genes in the cell, which may cause potential side effects. The fate manipulation of transplanted stem cells is equally important, and how to make the transplanted stem cells develop into specific neural cells according to the preconceived notion, survive stably and function for a longer period of time is one of the focuses and difficulties of current research (Dong et al., 2019; Berlet et al., 2022). The biggest obstacle to immune rejection is the recognition of HLA molecules on the surface of allogeneic stem cells. By knocking out the HLA-A, HLA-B, and HLA-C genes through CRISPR, iPSCs can make “universal” neural cells, and the incidence of rejection decreases dramatically when such cells are placed in non-human primate models (Xu et al., 2019). Whereas MSCs go about blocking T-cell activation by producing PD-L1 in large quantities, its immunogenicity is only 1/20 of that of fibroblasts (Li et al., 2021). And the sustainability of long-term efficacy is also an issue; animal experiments and preclinical studies are mostly short-term observations that lack adequate assessment and validation of the long-term efficacy and safety of the combination of stem cell and gene editing technologies in the treatment of AD, which needs to be determined by long-term follow-up studies (Rahimi Darehbagh et al., 2024) (Figure 2).

## 5 Future research directions and clinical application prospects

### 5.1 Technological innovation and optimization

**FIGURE 3:**
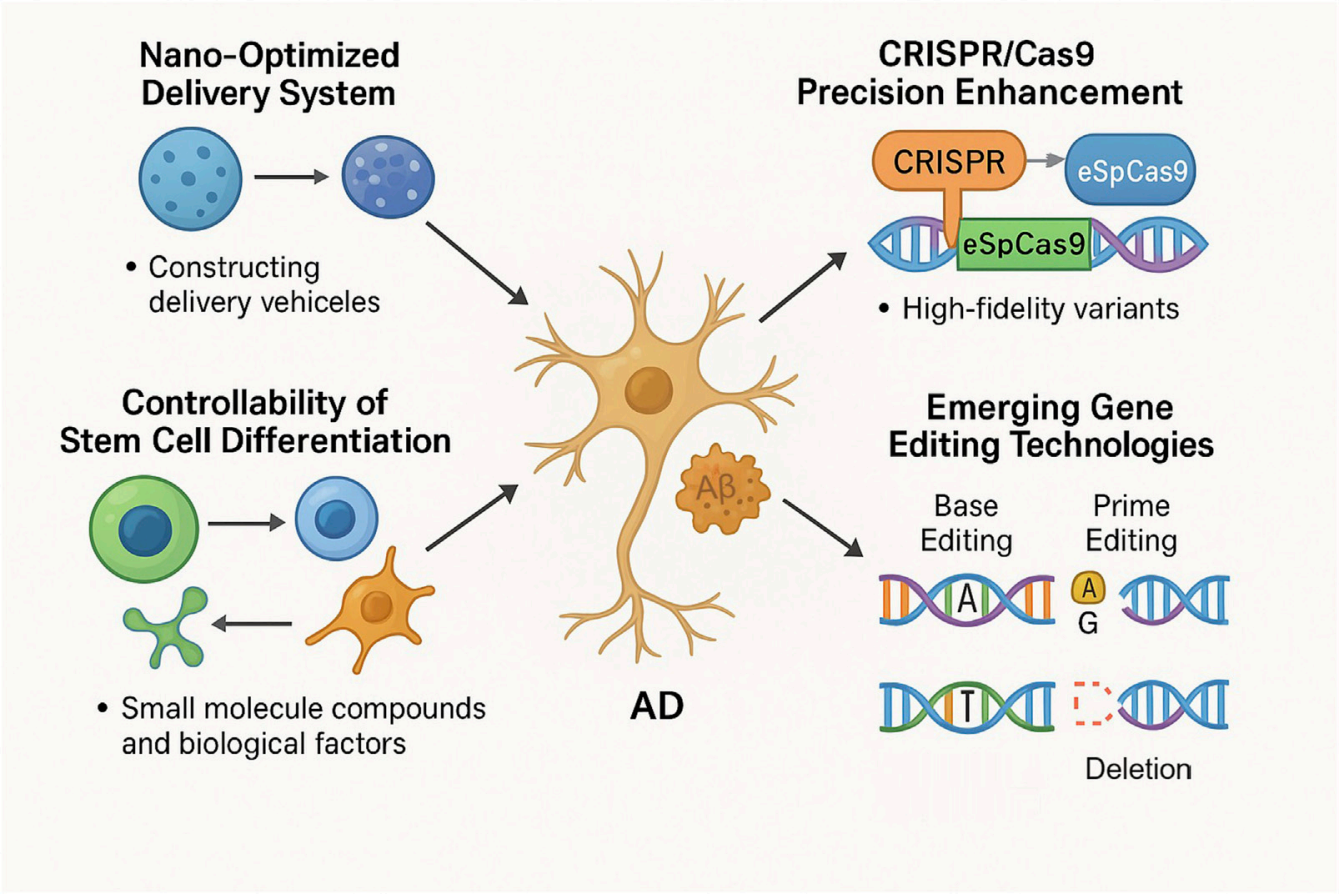
Innovative approaches to AD therapy include nano-optimized delivery systems, advanced CRISPR/Cas9 precision, controlled stem cell differentiation, and emerging non-cutting gene editing methods like base editing and prime editing. Nano-optimized systems enhance the efficiency of therapeutic delivery. Improvements in CRISPR/Cas9 variants (e.g., eSpCas9) optimize editing accuracy. Small molecule compounds and biokines enable precise control of stem cell differentiation. Base editing allows for precise, single-base corrections without DNA double-strand breaks, targeting specific point mutations in AD-related genes. Prime editing offers the flexibility to perform base substitutions, insertions, and deletions, addressing complex genetic mutations. These technologies collectively aim to remove Aβ plaques, repair neurons, and ameliorate AD pathology, providing safer and more effective therapeutic options.

Stem cell technology and gene editing technology have broad application prospects in AD therapy, and the development of optimized gene delivery systems is one of the hotspots in related research. New vectors constructed by nanotechnology can improve the delivery efficiency and targeting of tools such as CRISPR/Cas9, reduce off-target effects, and enhance therapeutic effects (Zhou et al., 2018). Structural modification of the Cas9 protein produces higher fidelity variants, such as SpCas9-HF1, eSpCas9, which are more precise in AD-related gene editing and more effective in correcting disease-causing variant mutations with less interference with non-target genes (Guo et al., 2019). These variants could not have emerged without AI-driven protein design: the AlphaFold2 predicted the Cas9-gRNA-DNA complex structure and guided mutation site screening, resulting in a significant decrease in the off-target rate of eSpCas9 compared to the wild type and greatly improving the specificity of gene editing (Jumper et al., 2021). The CRISPRoff algorithm, which uses machine learning to optimize gRNA sequences, has led to a significant increase in editing efficiency in PSEN1 gene editing, and the cleavage rate of non-target sites has been kept at a very low level, giving a new pathway to precision gene editing (Tyumentseva et al., 2023). On stem cell differentiation controllability, scientists try to regulate the direction of stem cell differentiation with small molecule compounds, biological factors, especially to make them accurately differentiate into neurons or glial cells, to generate specific types of cells on demand, to replace the damaged neuronal cells of AD patients, and to restore the function of the neural network (Hergenreder et al., 2024; Kaur et al., 2025). Non-cutting CRISPR systems, including base editing and Prime editing, have brought significant advances to AD treatment, with base editing being able to perform single base substitutions with precision without causing double-strand breaks, which is useful for correcting point mutations in AD-related genes, and this precision reduces the risk of unintentional genetic mutations, thus enhancing safety (Chen and Liu, 2023). Prime editing expands on this with its ability to do not only base substitutions, but also additions or deletions of small fragments, which gives versatile tools for dealing with the complex genetic background of AD, and these techniques give us a nuanced approach to gene correction that might reduce off-targeting and improve the accuracy of genetic modifications in neuronal cells (Anzalone et al., 2019). Base editing can correct specific pathogenic point mutations in the APP gene that cause amyloid plaque production, and Prime editing can be used to correct more complex mutations in the PSEN1 or PSEN2 genes (Fu et al., 2025). This ability to tailor genetic corrections so precisely opens the door to personalized medicine in AD treatment, allowing treatment options to be more tailored to an individual’s specific genetic characteristics (Figure 3). This technology not only improves efficiency, but also significantly improves safety, giving AD gene therapy a more reliable guarantee and perhaps helping AD patients reach better treatment choices before long.

### 5.2 Prospects for clinical application

In the field of personalized medicine, genetically tailored stem cell treatments are becoming feasible, raising the prospect of treating complex diseases such as AD with specialized gene editing to correct mutations in the APP, PSEN1 or PSEN2 genes (Wang S. W. et al., 2022). The use of stem cell technology to grow neural precursor cells that are genetically matched to the patient’s own genes has the advantage of increasing the relevance and effectiveness of the treatment, and is predicted to reduce immune rejection, thereby improving patient tolerance and compliance (Temple, 2023). The combination of gene therapy and stem cell transplantation may become the mainstay of AD treatment in the future, and some clinical trials have already begun to investigate the safety and efficacy of this combination. In animal models, anti-inflammatory or neurotrophic factor genes are added to stem cells using gene editing technology, and the genetically modified stem cells are then transplanted into the brains of mice modeled for AD (Zhou et al., 2023). Studies have shown that the combination therapy significantly improved cognitive function in mice and effectively reduced pathological changes in the brain, such as beta-amyloid deposition and neuroinflammatory responses (Huang D. et al., 2021). In terms of clinical translation, the combination therapy will probably be ready for clinical use in the next 5–10 years or so, as the technology matures and clinical trials begin, giving hope to patients with AD patients new hope.

### 5.3 Interdisciplinary collaboration and ethical considerations

Interdisciplinary collaboration is a major factor in the development of stem cell and gene editing technologies in AD, and experts in the fields of neurobiology, gene editing, and stem cell science need to join forces to deal with the difficulties. Stem cell scientists studying the microenvironment to promote stem cell growth and differentiation have realized the role of “gap-binding” proteins in stem cell differentiation, which has led to a new theoretical basis for related research (Wang Y. et al., 2022; Jin et al., 2025). Researchers have combined materials science and bioengineering to better differentiate stem cells into unique neural cell types, and gene editing experts have used a novel whole-brain gene editing technique developed by a team at the Hong Kong University of Science and Technology to alleviate AD pathology in a mouse model, demonstrating the therapeutic potential of gene editing for neurodegenerative diseases (Willerth and Sakiyama-Elbert, 2008). Multidisciplinary collaboration has greatly improved the accuracy of gene editing technology, and with computer simulations and the integration of multi-omics technologies, gene expression regulation has become more and more precise, and neurobiologists have further explored the physiology and pathology of neuronal cells, giving important pathomechanisms to support AD research (Liang et al., 2025). Neuroscience has merged with artificial intelligence technology, and researchers have gained a deeper understanding of the physiological and pathological mechanisms of neuronal cells. Ethical issues in the application of technology follow strict guidelines to ensure the rights and interests of patients and participants, especially in clinical trial sessions. Interdisciplinary teams must work together to develop and implement ethical guidelines, and in gene editing clinical trials, guidelines for informed consent are detailed, and participants should be fully informed of the purpose, risks, and benefits of the trial. Transparency and fair treatment of trial data are part of the ethical guidelines, which are important to ensure scientific validity and fairness of the trial (Coller, 2019; Rothschild, 2020). Informed consent states that participants should be fully informed of the purpose, risks, and benefits of the trial before joining the trial, and that disclosure and fair treatment of trial data is a matter of ethical protocols, which helps to maintain scientific rigor and fairness and prevents falsification and misuse of data, and that interdisciplinary collaborations and ethical guidelines will lead to the application of technological improvements that will bring new hope for the regulation of AD.

## 6 Conclusion and future perspectives

The combination of stem cell and gene editing technologies (especially CRISPR/Cas9) has brought a revolutionary approach to AD treatment, but the road from experimental success to clinical application is fraught with difficulties, which requires skillful updating of technology and deep ethical thinking. After exploring these technologies in depth, we feel that their true potential lies not just in their own capabilities, but in their collaboration with each other, where stem cells through gene editing can combine regenerative capabilities with precise genetic correction to give a comprehensive solution to the complexities of AD. CRISPR/Cas9-edited iPSCs have shown remarkable results in cutting Aβ and tau protein pathology as well as improving cognitive function in AD models, suggesting that they may be able to not only slow down symptoms but also alter the course of the disease. At the level of technological improvement, the development of nanotechnology has opened new doors for gene delivery, and things like lipid nanoparticles and polymer nanocarriers have received a lot of attention due to the efficiency and safety of their delivery efficacy, which has significantly scaled down off-target effects. As for the improvement of gene editing tools, the emergence of base editing and Prime editing brings hope for improving the accuracy and safety of editing, and may become the mainstream direction of future research. In terms of clinical application, we feel that personalized medicine is the future direction of AD treatment. With cutting-edge genetic screening technology as well as tailored gene editing and stem cell treatment protocols, we can formulate exclusive therapies for each patient’s different genetic characteristics to maximize treatment efficacy and minimize risk. A personalized approach not only improves the relevance and effectiveness of treatment, but also reduces immune rejection and improves patient tolerance and compliance. Ethical issues are particularly important in AD treatment research, especially germ cell editing for AD-related genes (APOE4). There is a great deal of controversy in this area right now, with China’s Regulations on Human Genetic Resources Management stating that gene editing of germ cells cannot be used for clinical purposes, and the U.S., while it doesn't prohibit basic research, it has to go through a rigorous institutional ethical review (regulated by the IRB along with the SAC). It is widely recognized in the scientific community that even if APOE4 gene editing could theoretically reduce the risk of AD, it could lead to “genetic enhancement” controversies that would impact social justice. That is why the formation of interdisciplinary ethics committees to evaluate risk-benefit ratios, the promotion of open public discussion, the improvement of clinical trial regulation, and the development of comprehensive ethical guidelines are necessary steps to ensure the responsible development of technology. During a clinical trial, every effort must be made to respect the autonomy of the participants and to ensure that they are aware of the purpose, risks and benefits of the trial, which is an ethical imperative and a key part of ensuring that the research is legitimate and socially acceptable. The combination of stem cell and gene editing technology has brought great expectations for AD treatment, but to realize this expectation, we need long-term thinking and efforts in technical and ethical aspects, in all aspects of society, interdisciplinary cooperation, and scientific researchers all over the world working together. We believe these technologies will help AD patients in the near future and open a new chapter in the treatment of neurodegenerative diseases.
